# Totally laparoscopic versus laparoscopy-assisted Billroth-I anastomosis for gastric cancer: a case–control and case-matched study

**DOI:** 10.1007/s00464-016-4872-3

**Published:** 2016-03-23

**Authors:** Mi Lin, Chao-Hui Zheng, Chang-Ming Huang, Ping Li, Jian-Wei Xie, Jia-Bin Wang, Jian-Xian Lin, Jun Lu, Qi-Yue Chen, Long-Long Cao, Ru-Hong Tu

**Affiliations:** Department of Gastric Surgery, Fujian Medical University Union Hospital, No. 29 Xinquan Road, Fuzhou, 350001 Fujian Province China

**Keywords:** Stomach neoplasms, Totally laparoscopic surgery, Modified delta-shaped gastroduodenostomy, Locally advanced

## Abstract

**Objective:**

To evaluate the safety, feasibility and clinical results of the modified delta-shaped gastroduodenostomy (MDSG) in totally laparoscopic distal gastrectomy (TLDG) for gastric cancer (GC).

**Methods:**

We performed a case–control and case-matched study enrolling 642 patients with GC undergoing laparoscopic distal gastrectomy with Billroth-I anastomosis from January 2011 to December 2014. TLDG with MDSG was performed in 158 patients (Group TL), and laparoscopy-assisted distal gastrectomy with circular anastomosis was performed in 484 patients (Group LA). One-to-one propensity score matching (PSM) was performed to compare the clinicopathological characteristics between the two groups.

**Results:**

Patients with smaller tumors or stage I cancer were more likely to receive TLDG (*P* < 0.05). In the propensity-matched analysis of 143 pairs, there were no differences in demographic and pathologic characteristics between groups (all *P* < 0.05). All patients successfully underwent laparoscopic radical distal gastrectomy. Before PSM, Group TL had more dissected lymph nodes (LNs), a longer time to first fluid diet and a longer postoperative length of stay than Group LA (all *P* < 0.05). After PSM, except for the fact that more dissected LNs were obtained in Group LA (*P* < 0.05), no difference was found in the intraoperative and postoperative outcomes between the groups (all *P* > 0.05). The postoperative complications were similar in both groups (all *P* > 0.05). Stratification analysis performed after PSM showed that in early GC, no difference was observed in intraoperative and postoperative outcomes between the groups (all *P* > 0.05). However, in locally advanced GC, Group TL had more dissected LNs and a higher rate of postoperative complications (both *P* < 0.05). Univariate analysis carried out in locally advanced cases after PSM showed that the body mass index (BMI), the method of digestive tract reconstruction and Charlson’s score were significant factors that affected postoperative morbidity (all *P* < 0.05). Multivariate analysis indicated that BMI was an independent risk factor for postoperative morbidity (*P* < 0.05).

**Conclusions:**

The MDSG in TLDG is safe and feasible for early GC; however, it should be chosen with caution in advanced GC, particularly in patients with a high BMI.

Totally laparoscopic radical gastrectomy has several advantages over laparoscopy-assisted surgery in terms of pulling, exposure, surgical field and minimally invasive effects [1–4]. For these reasons, this method has been gaining attention from laparoscopic surgeons. A new method for performing the intracorporeal Billroth-I anastomosis using only endoscopic linear staplers to complete the functional end-to-end anastomosis of the posterior walls of the gastric remnant and duodenum, referred to as the delta-shaped gastroduodenostomy (DSG), was first reported by Kanaya et al. [5]. The DSG procedure has been gaining acceptance in more centers because it is a relatively simple way to reduce the difficulty of the totally laparoscopic intracorporeal Billroth-I anastomosis [6–10]. Our institution has been utilizing this method since November 2012. As part of the implementation process, we proposed a modified DSG (MDSG) [11], which preliminary studies have demonstrated to be technically safe and feasible [12, 13]. However, most research on DSG currently includes retrospective studies, and the enrolled patients have mainly had early distal gastric cancer (GC). No studies focused on locally advanced distal GC have been reported. In addition, the use of propensity score matching (PSM) in retrospective studies can balance the covariates to control selective bias between groups [14] such that the results are more credible to provide better evidence. Thus, before conducting a prospective randomized controlled clinical trial, we performed a case–control and case-matched study using PSM to evaluate the safety, feasibility and clinical results of the MDSG in totally laparoscopic distal gastrectomy (TLDG) for GC, comparing it to laparoscopy-assisted distal gastrectomy (LADG) with circular anastomosis.

## Materials and methods

### Patients

**Fig. 1:**
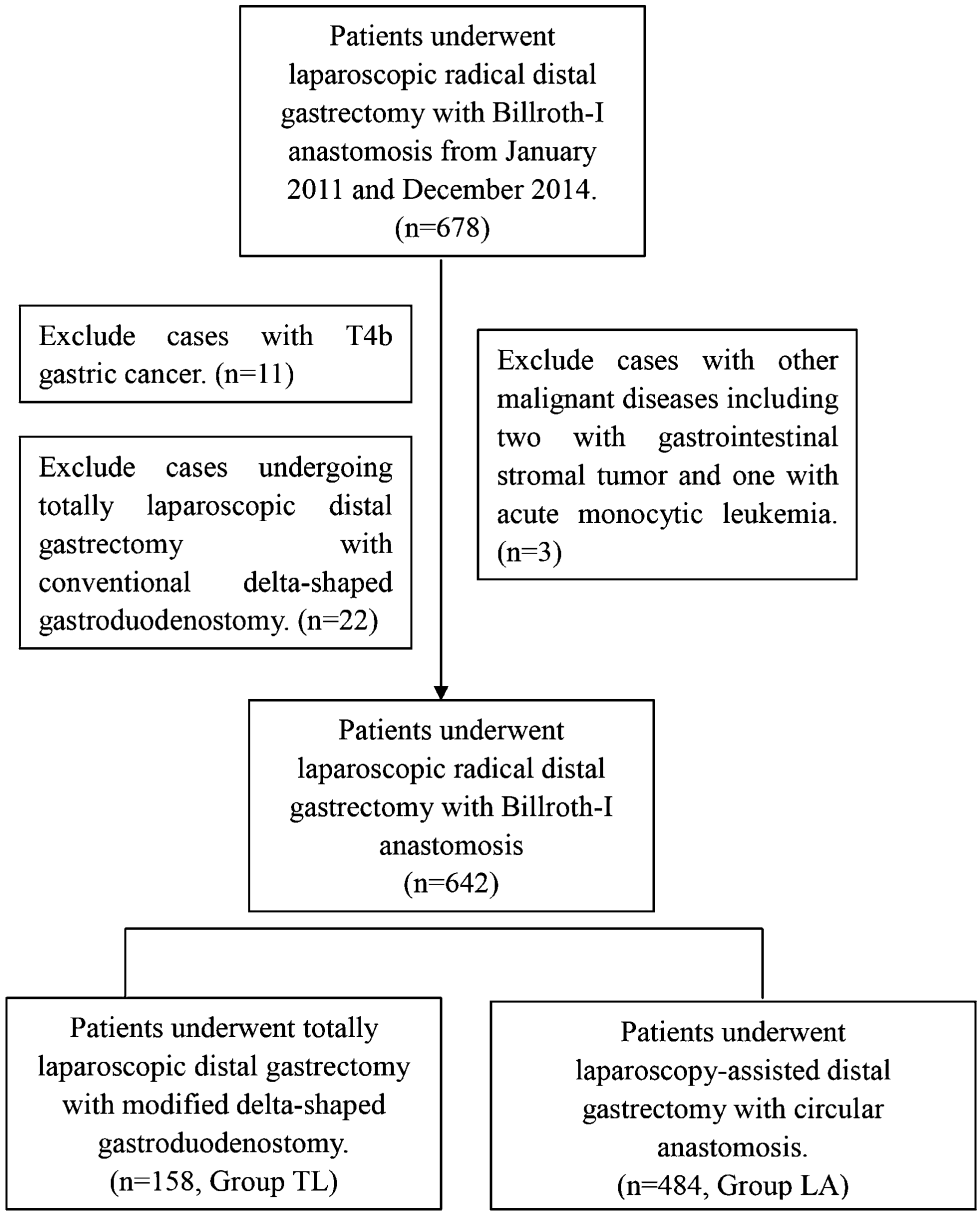
Enrollment of patients in the study

Between January 2011 and December 2014, 678 patients with primary distal GC underwent laparoscopic radical distal gastrectomy with Billroth-I anastomosis in the Department of Gastric Surgery, Fujian Medical University Union Hospital. Of these patients, three patients with other malignant diseases, 11 patients with T4b GC and 22 patients undergoing TLDG with conventional DSG were excluded. The remaining 642 patients were enrolled in the study. TLDG with MDSG was performed in 158 patients (Group TL), and LADG with a circular anastomosis was performed in 484 patients (Group LA) (Fig. 1). Distal GC was diagnosed preoperatively through analysis of endoscopic biopsy specimens. The pretreatment tumor site, depth of invasion, extent of lymph node (LN) metastasis and metastatic disease were assessed by endoscopy, computed tomography (CT), ultrasonography of the abdomen and/or chest radiography.

### Surgical procedures

All patients voluntarily chose laparoscopic surgery and provided written informed consent prior to surgery. All operations were performed by the same surgeon, who was proficient in laparoscopic surgery, having performed more than 2000 laparoscopic gastrectomy procedures. LN dissection was performed according to the guidelines of the Japanese Gastric Cancer Association [15]. The method of digestive tract reconstruction was according to the patient’s preference.

**Fig. 2:**
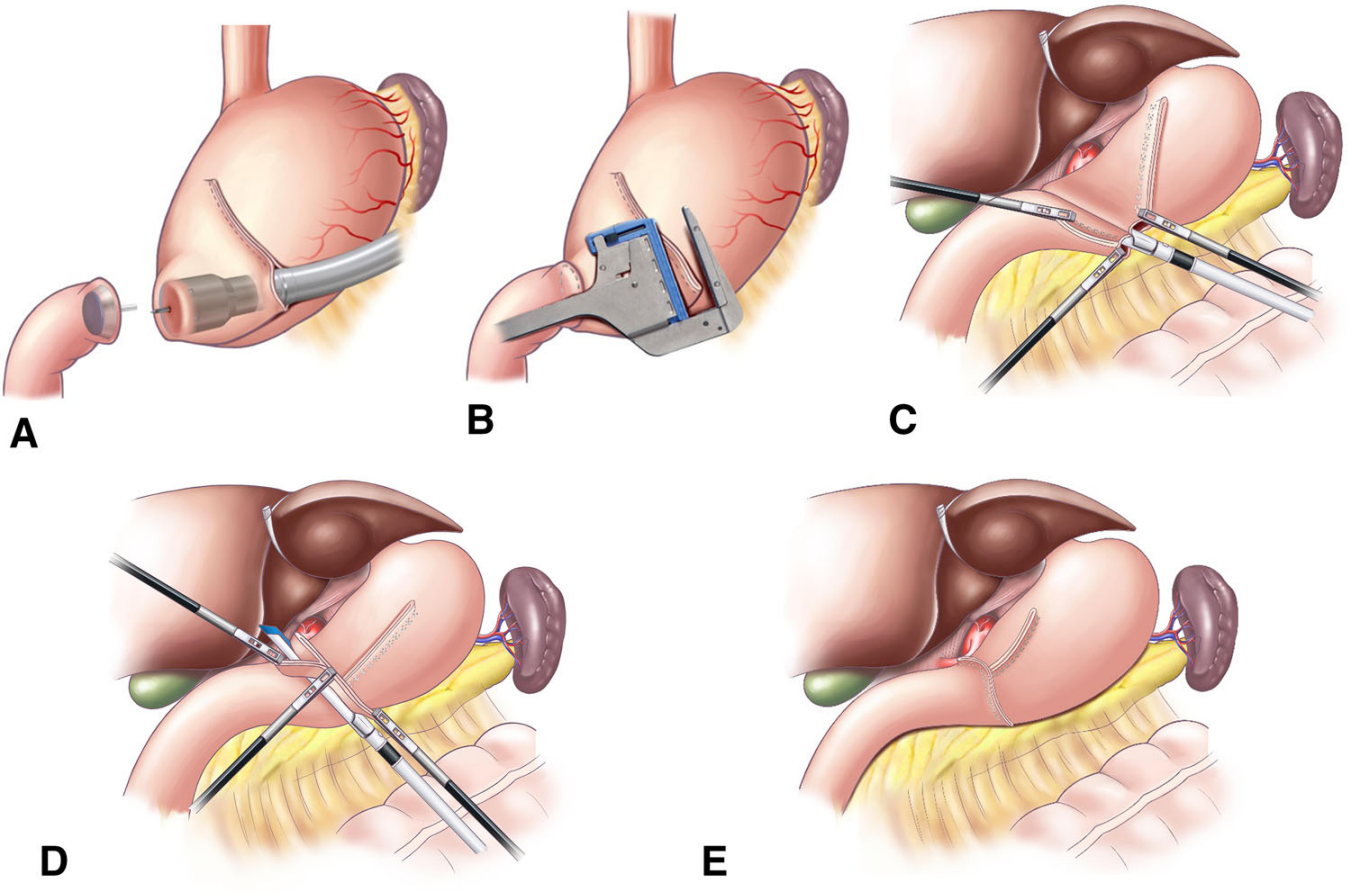
Procedures of laparoscopy-assisted circular anastomosis and modified delta-shaped gastroduodenostomy in totally laparoscopic distal gastrectomy. **A** A 28-mm *circular* stapler was inserted through the greater curvature to perform gastroduodenostomy. **B** A *linear* stapler was used to close the greater curvature of the stomach. **C** The stapler was positioned to join the posterior walls of the gastric remnant and duodenum together. **D** The completed involution of the common stab incision using the instruments of the surgeon and assistant with the blind angle of the duodenum being pulled up into the stapler. **E** The completed inverted T-shaped appearance of anastomosis

Laparoscopy-assisted circular anastomosis was conducted according to the traditional method. In our institution, an end-to-side Billroth-I procedure through 5–7 cm upper midline mini-laparotomy was performed. A 28-mm detachable anvil was inserted to the duodenal stump, and a purse string suture was tied over the purse string tying notch of the anvil. Then two Allen clamps were applied to the greater curvature of the stomach at a distance of 5 cm. After the gastric wall was incised between the two clamps, a linear stapler was used to divide the distal stomach and close the lesser curvature. Then a 28-mm circular stapler was inserted through the greater curvature to perform gastroduodenostomy (Fig. 2A). A linear stapler was used to close the greater curvature of the stomach (Fig. 2B).

The MDSG was carried out as described in the literature [11–13]. For this method, only endoscopic linear staplers were used under a totally laparoscopic approach. In brief, small incisions were made on the greater curvature of the remnant stomach and the posterior side of the duodenum. Following approximation of the posterior walls of the gastric remnant and duodenum, the forks of the stapler were closed and fired, creating a V-shaped anastomosis on the posterior wall (Fig. 2C). Then the instruments of the surgeon and the assistant directly grasped the tissue to efficiently accomplish the involution of the common stab incision. When the common stab incision was closed with the stapler, the blind angle of the duodenum was completely resected at the same time (Fig. 2D). The anastomosis appeared as an inverted T-shape (Fig. 2E).

### Data collection

A retrospective analysis was performed using a prospectively maintained comprehensive database to collect the clinicopathological and follow-up data for all patients. Charlson et al. [16] scoring system was used to evaluate preoperative comorbidity. Postoperative complications were graded according to the Clavien–Dindo scoring system [17]. Clinical and pathological staging were in accordance with the American Joint Committee on Cancer (AJCC) Seventh Edition of Gastric Cancer Tumor, Node, Metastasis (TNM) Staging [18]. The anastomosis was checked for leakage on postoperative days 7–9 by performing an upper gastrointestinal radiograph with diatrizoate meglumine as the contrast medium.

### Ethics statement

Institutional review board (IRB) of Fujian Medical Union Hospital approved this retrospective study. Written consent was given by the patients for their information to be stored in the hospital database and used for research.

### Statistical analysis

The statistical analyses were performed using the Statistical Package for the Social Sciences (SPSS), version 18.0 for Windows (SPSS Inc., Chicago, IL, USA). One-to-one PSM was performed between the two groups. Multiple-factor logistic regression models were used to calculate the propensity score for each patient; we imposed a caliper of 0.02 of the standard deviation of the logit of the propensity score. Patients in Group TL were individually matched to patients in Group LA according to the nearest neighbor matching principle and the non-replacement principle (i.e., a single case cannot be used multiple times). The measurement data are expressed as the means ± standard deviations. Categorical variables were analyzed using the Chi-square test or Fisher’s exact test, whereas continuous variables were analyzed using Student’s *t* test. To evaluate factors predictive of postoperative morbidity, multivariate analysis was performed using binary logistic multiple regression tests using dummy variables. *P* values <0.05 were considered statistically significant.

## Results

### Comparisons of clinicopathological characteristics between groups

**Fig. 3:**
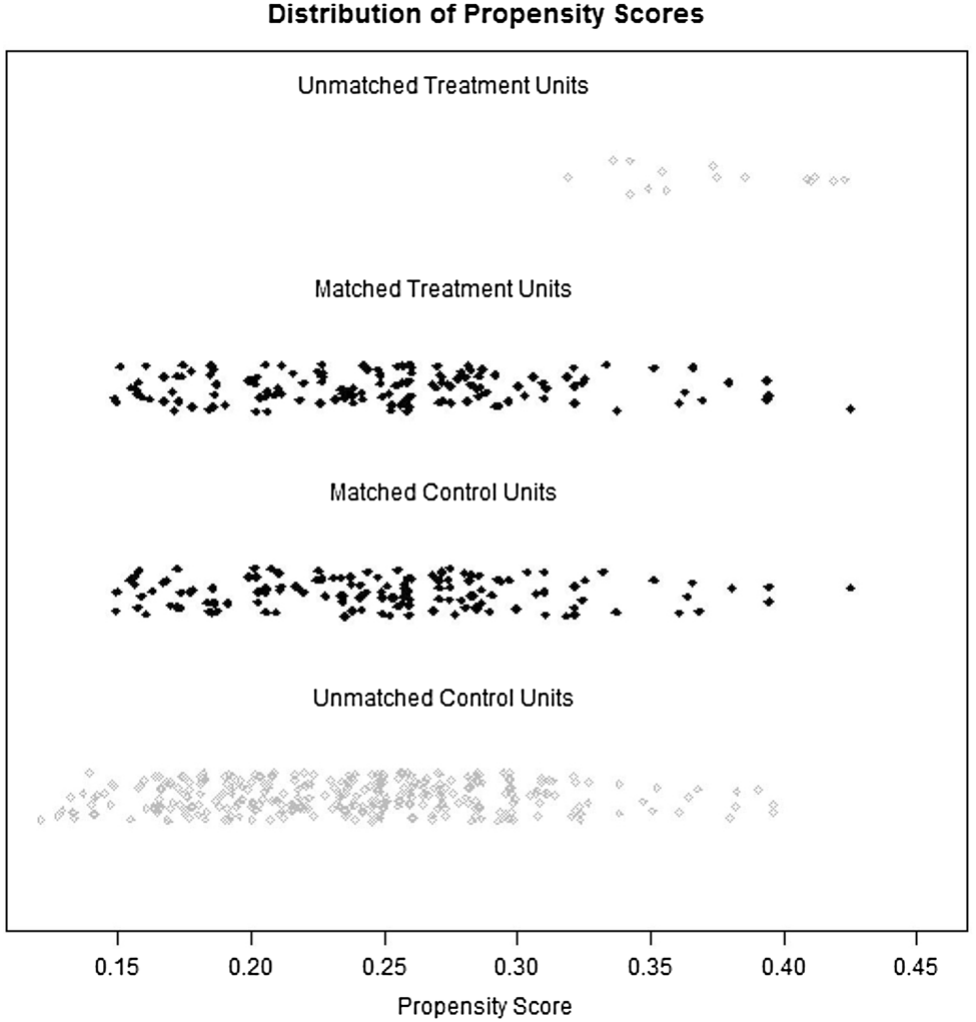
Distribution of propensity scores in the unmatched and matched units

The mean age was 59.7 ± 12.1 years (range 20–87 years), the mean body mass index (BMI) was 22.4 ± 3.2 kg/m^2^ (range 14.7–38.0 kg/m^2^), and the mean tumor size was 3.4 ± 2.0 cm (range 0.5–12.0 cm) in all 642 patients. Compared with Group LA before PSM, Group TL had a smaller tumor size and a larger proportion of early GC (all *P* < 0.05). Using one-to-one PSM according to age, gender, BMI, history of abdominal surgery, tumor size, tumor invasion (T), nodal metastasis (N) and TNM stage, 143 pairs from Groups TL and LA were matched (Fig. 3). There were no differences in demographic and pathologic characteristics between groups after PSM (all *P* < 0.05; Table 1).

**Table 1:** Comparisons of clinicopathological characteristics between groups [means ± standard deviations, *n* (%)]

Variables	Before PSM			After PSM		
	Group TL *n* = 158	Group LA *n* = 484	*P*	Group TL *n* = 143	Group LA *n* = 143	*P*
Age (year)	59.0 ± 13.1	59.9 ± 11.7	0.452	60.1 ± 12.7	59.4 ± 12.1	0.621
Gender			0.238			0.899
Male	102 (64.6)	337 (69.6)		100 (69.9)	102 (71.3)	
Female	56 (35.4)	147 (30.4)		43 (30.1)	41 (28.7)	
BMI	22.3 ± 3.2	22.5 ± 3.1	0.647	22.3 ± 3.1	22.5 ± 2.8	0.602
Chalson’s score			0.517			0.712
0–1	103 (65.2)	329 (68.0)		90 (62.9)	93 (65.0)	
≥ 2	55 (34.8)	155 (32.0)		53 (37.1)	50 (35.0)	
HB	127.1 ± 21.5	132.1 ± 66.4	0.362	127.2 ± 22.2	127.4 ± 26.1	0.951
History of abdominal operation			0.056			0.125
No	125 (79.1)	414 (85.5)		122 (85.3)	112 (78.3)	
Yes	33 (20.9)	70 (14.5)		21 (14.7)	31 (21.8)	
Tumor size(cm)	3.2 ± 1.9	3.5 ± 2.0	0.043	3.2 ± 1.9	3.6 ± 2.0	0.053
pT			0.004			0.239
T1	73 (46.2)	202 (41.7)		65 (45.4)	60 (42.0)	
T2	25 (15.8)	57 (11.8)		22 (15.4)	19 (13.3)	
T3	46 (29.1)	120 (24.8)		42 (29.4)	38 (26.5)	
T4	14 (8.9)	105 (21.7)		14 (9.8)	26 (18.2)	
pN			0.129			0.068
N0	78 (49.4)	247 (51.0)		72 (50.3)	75 (52.4)	
N1	29 (18.4)	70 (14.5)		28 (19.6)	16 (11.2)	
N2	28 (17.7)	64 (13.2)		25 (17.5)	21 (14.7)	
N3	23 (14.6)	103 (21.3)		18 (12.6)	31 (21.7)	
pTNM			0.036			0.130
IA	54 (34.2)	173 (35.7)		48 (33.5)	53 (37.0)	
IB	23 (14.6)	47 (9.7)		23 (16.1)	11 (7.7)	
IIA	24 (15.2)	53 (11.0)		22 (15.4)	20 (14.0)	
IIB	16 (10.1)	51 (10.5)		15 (10.5)	13 (9.1)	
IIIA	16 (10.1)	43 (8.9)		12 (8.4)	11 (7.7)	
IIIB	20 (12.7)	61 (12.6)		18 (12.6)	20 (14.0)	
IIIC	5 (3.2 %)	56 (11.6)		5 (3.5)	15 (10.5)	
With pyloric obstruction			0.184			0.367
No	157 (99.4)	470 (97.1)		142 (99.3)	139 (97.2)	
Yes	1 (0.6)	14 (2.9)		1 (0.7)	4 (2.8)	
With hemorrhage			0.324			0.670
No	145 (91.8)	45 (94.0)		130 (90.9)	132 (92.3)	
Yes	13 (8.2)	29 (6.0)		13 (9.1)	11 (7.7)	

*PSM* propensity score matching, *Group TL* patients underwent totally laparoscopic distal gastrectomy with modified delta-shaped gastroduodenostomy, *Group LA* patients underwent laparoscopy-assisted distal gastrectomy with circular anastomosis, *BMI* body mass index, *HB* hemoglobin B, *pT* pathologic T staging, *pN* pathologic N staging, *pM* pathologic M staging, *pTNM* pathologic tumor, noes and metastasis staging

### Comparisons of surgical outcomes between groups

All patients successfully underwent laparoscopic radical distal gastrectomy, with only few curable complications occurred and no operation-related death during the perioperative period, and all patients were uneventfully discharged. For all 642 patients, the mean number of harvested LNs was 32.8 ± 10.7 per patient, the mean operation time was 155.1 ± 42.7 min and the media total blood loss was 57.5 ± 40.8 mL. Before PSM, Group TL had more dissected LNs, a longer time to the first fluid diet and a longer postoperative length of stay than Group LA (all *P* < 0.05). The operation time, total blood loss, time to first flatus and time to soft diet were not significantly different between the two groups (all *P* > 0.05). After PSM, no difference was found in the intraoperative and postoperative outcomes between the two groups (all *P* > 0.05) except for the fact that more dissected LNs were obtained in Group LA (*P* < 0.05; Table 2).

**Table 2:** Comparisons of surgical outcomes between groups

Variables	Before PSM			After PSM		
	Group TL *n* = 158	Group LA *n* = 484	*P*	Group TL *n* = 143	Group LA *n* = 143	*P*
No. of retrieved LNs	35.7 ± 11.4	31.9 ± 10.3	0.000	35.7 ± 11.5	31.7 ± 9.6	0.002
Operation time	154.4 ± 30.1	155.6 ± 46.2	0.776	154.9 ± 30.3	153.9 ± 46.0	0.826
Blood loss	51.1 ± 30.9	61.6 ± 78.3	0.121	51.1 ± 31.4	63.0 ± 101.2	0.195
Day of first flatus	3.9 ± 1.4	4.0 ± 1.6	0.293	3.9 ± 1.5	4.1 ± 1.6	0.140
Day of first fluid diet	5.1 ± 1.8	4.7 ± 1.6	0.008	5.2 ± 1.9	4.9 ± 1.7	0.128
Day of first semifluid diet	8.0 ± 2.6	7.9 ± 1.9	0.589	8.0 ± 2.6	8.1 ± 2.1	0.765
Hospital stay	12.7 ± 7.2	11.5 ± 5.4	0.038	12.8 ± 7.4	11.9 ± 6.5	0.285

*PSM* propensity score matching, *Group TL* patients underwent totally laparoscopic distal gastrectomy with modified delta-shaped gastroduodenostomy, *Group LA* patients underwent laparoscopy-assisted distal gastrectomy with circular anastomosis, *LNs* lymph nodes

### Comparisons of postoperative complications between groups

The overall complication rate of all patients before and after PSM was 11.4 and 12.6 %, respectively. Postoperative complications were graded according to the Clavien–Dindo scoring system. The III–IV complications in Group TL were as follows: three patients experienced pulmonary infection and were all treated in the intensive care unit (ICU); two experienced celiac infection and received puncture and drainage with CT guidance; two experienced anastomotic leakage and had a nasojejunal feeding tube placed under X-ray; one experienced abdominal hemorrhage (not including anastomotic bleeding) with exploratory laparotomy treatment to achieve hemostasis; one experienced septicemia and was treated in the ICU; and one experienced inflammatory intestinal obstruction and was treated with endoscopic exploration. The III–IV complications in Group LA were as follows: five patients experienced pulmonary infection, four of whom were treated in the ICU and one of whom received drainage of pleural puncture under local anesthesia; five patients had a nasojejunal feeding tube placed under X-ray, including three anastomotic leakages and two gastric atony diseases; two experienced anastomotic bleeding, one of whom underwent exploratory laparotomy and the one of whom underwent endoscopic exploration to achieve hemostasis; two experienced abdominal hemorrhage (not including anastomotic bleeding), both of whom were treated with exploratory laparotomy to achieve hemostasis; one experienced celiac infection and underwent puncture and drainage by CT guidance; and one experienced an incision infection and underwent re-suturing under general anesthesia. All patients with postoperative complications were cured and discharged. The postoperative complications were similar in the groups before and after PAM (all *P* > 0.05); anastomosis-related complications were also comparable (all *P* > 0.05; Table 3).

**Table 3:** Comparisons of postoperative complications between groups

Variables	Before PSM			After PSM		
	Group TL *n* = 158	Group LA *n* = 484	*P*	Group TL *n* = 143	Group LA *n* = 143	*P*
Complications grade^a^			0.156			0.309
0	134 (84.8 %)	435 (89.9 %)		121 (84.6 %)	129 (90.2 %)	
I–II	14 (8.9 %)	33 (6.8 %)		12 (8.4 %)	9 (6.3 %)	
Pulmonary infection	3	8		2	2	
Celiac infection	1	7		1	3	
Urinary infection	2	0		1	0	
Arrhythmia	0	1		0	1	
Lymphatic leakage	3	4		3	2	
Lower incomplete gastrointestinal obstruction	1	0		1	0	
Incision infection	0	3		0	0	
Anastomotic leakage	3	2		3	0	
Anastomotic hemorrhage	0	2		0	0	
Gastric atony	1	6		1	1	
III–IV	10 (6.3 %)	16 (3.3 %)		10 (7.0 %)	5 (3.5 %)	
Pulmonary infection	3	5		3	3	
Septicemia	1	0		1	0	
Inflammatory intestinal obstruction	1	0		1	0	
Celiac infection	2	1		2	0	
Incision infection	0	1		0	0	
Abdominal hemorrhage (not including anastomotic bleeding)	1	2		1	0	
Anastomotic leakage	2	3		2	0	
Anastomotic bleeding	0	2		0	2	
Gastric atony	0	2		0	0	
Complication rate	15.2 %	10.1 %	0.082	15.4 %	9.8 %	0.154
Anastomosis-related complications	6 (3.8 %)	17 (3.5 %)	0.867	6 (4.2 %)	3 (2.1 %)	0.501
Anastomotic leakage	5 (3.2 %)	5 (1.0 %)	0.131	5 (3.5 %)	0 (0.0 %)	0.060
Anastomotic hemorrhage	0 (0.0 %)	4 (0.8 %)	0.577	0 (0.0 %)	2 (1.4 %)	0.498
Gastric atony	1 (0.6 %)	8 (1.7 %)	0.577	1 (0.7 %)	1 (0.7 %)	1.000
Anastomotic stricture	0	0	–	0	0	–

*PSM* propensity score matching, *Group TL* patients underwent totally laparoscopic distal gastrectomy with modified delta-shaped gastroduodenostomy, *Group LA* patients underwent laparoscopy-assisted distal gastrectomy with circular anastomosis

^a^Postoperative complications were graded according to the Clavien-Dindo scoring system

#### Stratification analysis of surgical outcomes between groups

Stratification analysis by early or locally advanced stage was performed for all cases after PSM. In the early GC, no difference in intraoperative and postoperative outcomes was found between the groups (all *P* > 0.05). However, in locally advanced GC, Group TL had more dissected LNs and a higher rate of postoperative complications (both *P* < 0.05; Table 4).

**Table 4:** Stratification analysis of surgical outcomes between groups

Variables	Early GC			Locally advanced GC		
	Group TL *n* = 65	Group LA *n* = 60	*P*	Group TL *n* = 78	Group LA *n* = 83	*P*
No. of retrieved LNs	32.5 ± 11.3	29.4 ± 8.2	0.088	38.2 ± 11.1	33.3 ± 10.1	0.004
Operation time	153.5 ± 30.7	144.4 ± 33.6	0.129	156.1 ± 30.2	160.3 ± 52.0	0.544
Blood loss	52.0 ± 27.4	51.7 ± 31.0	0.955	50.4 ± 34.5	70.6 ± 128.2	0.191
Day of first flatus	3.7 ± 1.0	3.9 ± 1.2	0.255	4.0 ± 1.7	4.3 ± 1.8	0.323
Day of first fluid diet	4.9 ± 1.0	4.6 ± 0.9	0.159	5.4 ± 2.3	5.0 ± 2.1	0.237
Day of first semifluid diet	7.5 ± 1.3	7.9 ± 1.8	0.130	8.4 ± 3.3	8.2 ± 2.2	0.624
Hospital stay	12.17 ± 5.2	11.0 ± 3.3	0.176	13.3 ± 8.8	12.5 ± 7.9	0.535
Operative complication	7(10.8 %)	7(11.7 %)	0.874	15(19.2 %)	7(8.4 %)	0.046
Anastomosis-related complications	1(1.5 %)	1(1.7 %)	1.000	5(6.4 %)	2(2.4 %)	0.265
Anastomotic leakage	1(1.5 %)	0(0.0 %)	1.000	4(5.1 %)	0(0.0 %)	0.053
Anastomotic hemorrhage	0(0.0 %)	1(1.7 %)	0.480	0(0.0 %)	1(1.2 %)	1.000
Gastric atony	0	0	–	1(1.3 %)	1(1.2 %)	1.000
Anastomotic stricture	0	0	–	0	0	–

*GC* gastric cancer, *Group TL* patients underwent totally laparoscopic distal gastrectomy with modified delta-shaped gastroduodenostomy, *Group LA* patients underwent laparoscopy-assisted distal gastrectomy with circular anastomosis, *LNs* lymph nodes

#### Risk factors of postoperative complications in locally advanced GC

An analysis of predictable risk factors associated with postoperative complications was performed in patients with locally advanced GC after PSM. Univariate analysis showed that BMI, the method of digestive tract reconstruction and Charlson’s score were significant factors that affected postoperative morbidity (all *P* < 0.05). The factors with *P* < 0.05 in the univariate analysis were included in the multivariate logistic regression analysis. BMI was identified as an independent risk factor for postoperative morbidity (*P* < 0.05; Table 5).

**Table 5:** Risk factors of postoperative complications in locally advanced gastric cancer

Variables	Postoperative complications		Univariate analysis	Multivariate analysis		
	Yes	No	*P*	OR	95 % CI	*P*
BMI	24.4 ± 3.2	22.0 ± 2.9	0.000	1.278	1.087–1.501	0.003
Digestive tract reconstruction			0.046	2.741	0.993–7.562	0.052
TLDG	15 (19.2 %)	63 (80.8)				
LADG	7 (8.4 %)	76 (91.6 %)				
Chalson’s score			0.027	2.366	0.812–6.893	0.114
0–1	16 (11.2 %)	127 (88.8 %)				
≥ 2	6 (33.3 %)	12 (66.7 %)				

*BMI* body mass index, *OR* odds ratio, *CI* confidence interval, *TLDG* totally laparoscopic distal gastrectomy with modified delta-shaped gastroduodenostomy, *LADG* laparoscopy-assisted distal gastrectomy with circular anastomosis

## Discussion

Laparoscopic distal gastrectomy has been the standard treatment for early distal GC [19, 20]. Several large-sample and multicenter retrospective studies have also demonstrated the safety and feasibility of a laparoscopic technique for locally advanced distal GC [21]. To date, the main method of reconstruction used in laparoscopic distal gastrectomy is the Billroth-I circular anastomosis through a small incision in the abdominal wall. However, since Kanaya et al. [5] first proposed the DSG in TLDG in 2002, it has grown in popularity because of its relative simplicity and superior laparoscopic surgical field. Multiple researchers have confirmed these clinical results. In the single-arm study of Kanaya et al. [22], the clinical data of 100 patients undergoing DSG were analyzed. The results showed that the method was safe, simple and less invasive. In the comparative studies between DSG and LADG with a circular anastomosis, the majority of results showed that there was no difference in the surgical time and postoperative complication rate [1, 8, 23], and the long-term outcomes were also comparable [10]. DSG was considered to be less invasive [1, 3], especially in obese patients [8, 24]. Previous studies in our center revealed that the MDSG had similar clinical outcomes compared with conventional DSG and could shorten the time of anastomosis [12, 13].

Unfortunately, there is still a lack of advanced evidence supporting the efficacy of the DSG. Thus, before DSG can become a universally applicable technique for most patients with GC, a retrospective analysis of the clinical outcomes using a large-scale data set with appropriate statistical methods and proper study design is required. In this study, we compared the MDSG in TLDG with the commonly used circular anastomosis in LADG. To control for selection bias in this retrospective study, the PSM method was used to balance the confounding variables; this improved the comparability of the two groups and made the results more authentic and reliable [14, 25]. Before PSM, the tumor size, T and TNM stage in the two groups were significantly different; after PSM, there was no significant difference between groups, resulting in a good balance. After matching, no difference was found in the short-term outcomes between the two groups except that more dissected LNs were obtained in Group LA.

In regard to the postoperative complications, there are varied morbidity rates in laparoscopic radical gastrectomy for GC. Many studies have reported that morbidity rates for laparoscopic surgery range from 11.6 to 18.7 % [20, 26, 27], although some centers have reported rates of 24.9–42.6 % [28–30]. In our study, the overall complication rate of all patients before and after PSM was 11.4 and 12.6 %, respectively, similar to the literature. However, over 10 % of complication rate should not be neglected. It would have a certain impact on the postoperative quality of life. Therefore, we should take measures to prevent and minimize the morbidity rate. For example, before operation, active management of patients and aggressive treatment of comorbidities are required to improve the physical condition of patient; during operation, delicate surgical manipulation is required to minimize surgical trauma and hemorrhage, and the stapler should be used correctly and skillfully; and after operation, the patient should obtain close observation and nursing, the tubes should be kept patency and early intervention should be performed when abnormal clinical manifestations occurred.

In addition, because this is a new method, the patients with early distal GC were the main research subjects in DSG studies. Enrolled patients with I stage GC represented more than 85 % of all patients. Patients with locally advanced GC were less common, and most of them had stage II or stage IIIA GC [5, 22, 23]. Stage IIIB and IIIC diseases were rarely reported. Patients with stage I GC were also the main research subjects in the study of MDSG [12, 13]. However, in most countries in the world, with the exception of Japan and Korea, most patients with GC are diagnosed with advanced disease. Whether TLDG with DSG is suitable for locally advanced GC remains a question for further discussion.

Based on previous studies, we believed that TLDG with MDSG in early GC was safe and feasible. With the accumulation of clinical experience, we have also gradually attempted to perform TLDG with MDSG in locally advanced distal GC for exploratory research. Therefore, patients with locally advanced GC accounted for more than 50 % of this study sample. Stratification analysis showed that the short-term outcomes in early GC were similar between the two groups. However, in locally advanced GC, the postoperative complication rate in Group TL was higher than that in Group LA. Although there was no significant difference between the groups in terms of anastomotic leakage, there were four cases of anastomotic leakage in Group TL, whereas there were no instances of anastomotic leakage in Group LA; thus, this problem should be taken seriously. Because a suitably sized remnant stomach and duodenum should be produced to ensure not only R0 tumor resection but also appropriate anastomotic tension, DSG might be more suitable for patients with early or locally advanced GC in a relatively early stage. The results before PSM in this study also demonstrated that Group TL had smaller tumors and a larger proportion of early GC than Group LA. Considering the risk factors identified for the postoperative complications, patients with high BMI in locally advanced GC may increase the difficulty of the operation. Thus, the risk of surgery and the rates of postoperative complications were increased under those conditions. This suggests that MDSG in TLDG should be carefully chosen in locally advanced distal GC, especially for patients with a high BMI. During surgery, attention should be paid to the placement of an intraoperative reinforcing suture, which, along with perioperative active management, might help prevent postoperative complications.

In conclusion, MDSG in TLDG is safe and feasible in the treatment of early distal GC, but its indications should receive careful consideration. More care should be taken in making treatment decisions in locally advanced distal GC, especially in patients with a high BMI. This study used PSM to reduce selection bias, which made the results more reliable. However, this was a single-center retrospective study, and there are still some limitations. Some results, for instance, whether the LN retrieval was less in Group LA comparing with Group TL is true, need large-sample or prospective, multicenter randomized studies to provide more accurate evidence.
